# The Genetic Characteristics and Carbapenem Resistance Mechanism of ST307 *Klebsiella pneumoniae* Coharbouring *bla*_CMY-6_, *bla*_OXA-48_, and a Truncated *bla*_NDM-1_

**DOI:** 10.3390/antibiotics11111616

**Published:** 2022-11-13

**Authors:** Qiucheng Shi, Xinhong Han, Qin Huang, Yan Meng, Ping Zhang, Zhengan Wang, Huangdu Hu, Yan Jiang, Xiaoxing Du, Yunsong Yu

**Affiliations:** 1 Department of Infectious Diseases, Sir Run Run Shaw Hospital, Zhejiang University School of Medicine, Hangzhou 310020, China; 2 Key Laboratory of Microbial Technology and Bioinformatics of Zhejiang Province, Hangzhou 310020, China; 3 Regional Medical Center for National Institute of Respiratory Diseases, Sir Run Run Shaw Hospital, Zhejiang University School of Medicine, Hangzhou 310020, China; 4 Department of Intensive Care Unit, The Affiliated Hospital of Medical School, Ningbo University, Ningbo 315020, China; 5 Department of Clinical Laboratory, Zhejiang Hospital, Zhejiang University School of Medicine, Hangzhou 310030, China

**Keywords:** sequence type 307, carbapenem-resistant, *Klebsiella pneumoniae*, truncated NDM-1, *bla*_OXA-48_, *bla*_CMY-6_

## Abstract

Carbapenem-resistant *Klebsiella pneumoniae* (CRKP) is a common nosocomial pathogen causing severe infectious diseases, and ST307 CRKP is an emerging clone. In this study, we collected five ST307 CRKP isolates, evaluated their antimicrobial susceptibility using microbroth dilution, and their clonality and population structure by PFGE, cgMLST, and SNP-based phylogenetic analysis. Then, the genome characteristics, such as antimicrobial resistance genes and plasmid profiles, were studied by subsequent genomic analysis. The plasmid transfer ability was evaluated by conjugation, and the carbapenem resistance mechanism was elucidated by gene cloning. The results showed that all five ST307 CRKP isolates harboured *bla*_CMY-6_, *bla*_OXA-48_, and *bla*_NDM-1_; however, the end of the *bla*_NDM-1_ signal peptide was interrupted and truncated by an IS*10* element, resulting in the deactivation of carbapenemase. The ST307 isolates were closely related, and belonged to the globally disseminated clade. *bla*_OXA-48_ and *bla*_NDM-1_ were located on the different mobilisable IncL/M- and IncA/C2-type plasmids, respectively, and either the pOXA-48 or pNDM-1 transconjugants were ertapenem resistant. Gene cloning showed that *bla*_CMY-6_ could elevate the MICs of carbapenems up to 64-fold and was located on the same plasmid as *bla*_NDM-1_. In summary, ST307 is a high-risk clone type, and its prevalence should be given additional attention.

## 1. Introduction

*Klebsiella pneumoniae* is responsible for multiple human infectious diseases, such as abdominal infections, respiratory tract infections, and bloodstream infections, which result in severe morbidity and mortality [1]. Carbapenems are commonly used for the treatment of severe bacterial infections and are considered ‘last-resort’ antibiotics for using against multidrug-resistant (MDR) Gram-negative bacteria [2]. However, with extensive use, the rate of development of resistance to carbapenems has accelerated rapidly in recent decades [3], and nationwide surveillance has shown that the prevalence of carbapenem-resistant *K. pneumoniae* (CRKP) has reached 27.5% in China (http://chinets.com (accessed on 23 September 2022)).

It is well known that the leading carbapenem resistance mechanism is the production of carbapenemases, which commonly include KPC, NDM, OXA, IMP, and VIM [4]. Previous epidemiological research showed that *bla*_KPC_ was the most widely disseminated carbapenemase in China, accounting for 74% of CRKP strains, whereas 17% of CRKP strains were *bla*_NDM_ positive [5]. Recently, an increased prevalence of *bla*_OXA-48_ with relatively weak carbapenem antibiotic hydrolysis ability has been reported [6]. Sequence type (ST) 11, closely related to the globally spreading epidemic clonal group (CG) 258, is the most common clone of CRKP prevailing in China and is closely associated with the carbapenemase KPC; meanwhile, other CRKP clone types, such as CG15, ST307, and ST147, have recently emerged globally [7,8].

In this study, we found five ST307 CRKP isolates that coharboured *bla*_CMY-6_, *bla*_OXA-48_, and a truncated *bla*_NDM-1_. Antimicrobial susceptibility testing (AST), clonality analysis, and genomic analysis were performed to identify the phylogenetic relationship and genomic characteristics of ST307 CRKP. The conjugation assay showed that either the pOXA-48 or pNDM-1 transconjugants were resistant to ertapenem, and even pNDM-1 had a truncated *bla*_NDM-1_. In addition, long-read sequencing analysis and gene cloning experiments were performed to explore the carbapenem resistance mechanism among these isolates.

## 2. Results

### 2.1. Clonality Analysis and Population Structure of ST307

In this study, the AST showed that the isolates were resistant to aztreonam, ceftazidime, imipenem, meropenem, and ertapenem; as a result, these isolates were considered CRKP. Five CRKP isolates all belonged to ST307; furthermore, their pulsed-field gel electrophoresis (PFGE) patterns were indistinguishable, and there were fewer than three different bands among these isolates. Similarly, core genome multilocus sequence typing (cgMLST) analysis indicated that the average core gene allele difference of these isolates was 6.8 ± 3.6, which indicated that they were closely related (Figure S1A,B).

**Figure 1:**
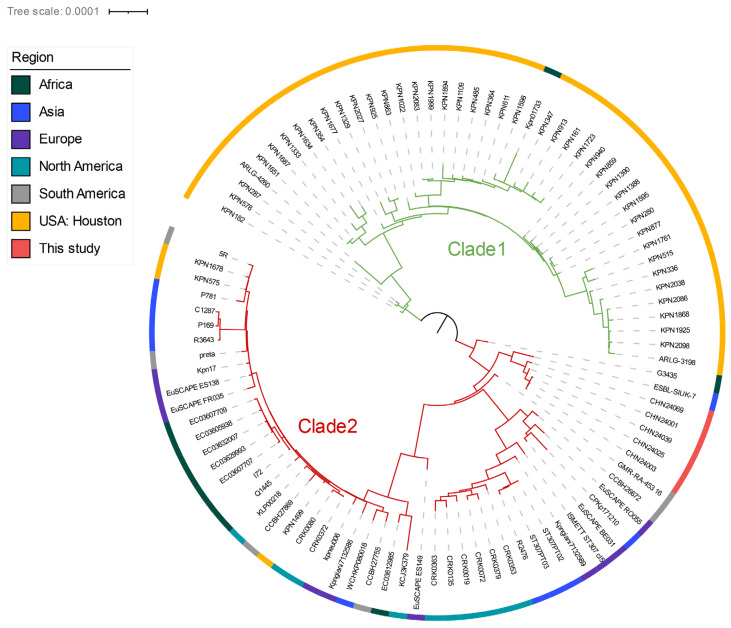
Population structure of ST307 worldwide along with the ST307 isolates in this study. Maximum-likelihood inferred phylogeny of ST307 isolates using strain C234 as a reference for core gene alignment with midpoint rooting. The branch label background corresponds to clades 1 and 2, and the outer ring indicates the region where the isolate was collected.

According to Shropshire et al., the global ST307 population could be divided into four clades. The Houston-based ST307 clades belonged to clades 1, 3, and 4; however, the globally disseminated ST307 was located in clade 2 [9]. ST307 had a unique endemic spread in Houston, and we tried to identify the cluster location of ST307 in this study within the international population. For this purpose, we randomly selected the globally distributed ST307 genome from a public database, and the phylogenetic tree could be divided into two clades. The results showed that the Houston-based isolates belonged to clade 1, whereas the isolates in this study belonged to clade 2, which is a globally disseminated clade (Figure 1).

### 2.2. Antimicrobial Resistance Genes in the Isolates

All five isolates were positive for *bla*_CMY-6_, *bla*_NDM-1_, *bla*_OXA-48_, *bla*_SHV-106_, and *bla*_TEM-1_, and two of them, CHN14001 and CHN24069, were additionally *bla*_CTX-M-15_ and *bla*_OXA-1_ positive (Table 1). However, we found that *bla*_NDM-1_ was truncated by IS*10* at amino acid position 25, and the residues from position 27 to 270 of the intact amino acids were completely identical (Figure S2). Because fragment 1–28 is considered as a signal peptide (UniProt entry C7C422), the insertion at this site resulted in inactivation of the *bla*_NDM-1_ gene. The results of the gene cloning experiment indicated that truncation of *bla*_NDM-1_ led to carbapenem susceptibility, whereas the clone with intact *bla*_NDM-1_ was resistant.

### 2.3. Difference in pNDM-1 and pOXA-48 among These Isolates

**Figure 2:**
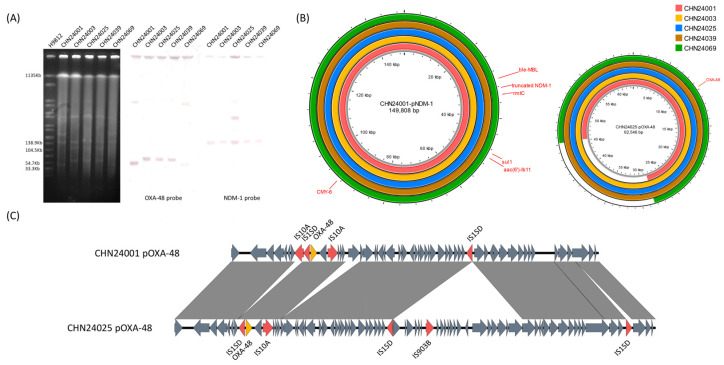
(**A**) The S1-digested plasmid DNA and the Southern blotting of CHN24001, CHN24003, CHN24025, CHN24039, and CHN24069 with the *bla*_NDM-1_ and *bla*_OXA-48_ probes. (**B**) Comparison of pNDM-1 and pOXA-48 among these isolates. The red ring represents CHN24001, the yellow ring represents CHN24003, the blue ring represents CHN24025, the brown ring represents CHN24039, and the green ring represents CHN24069. Antimicrobial resistance genes are labelled in the figures. (**C**) The comparison of pOXA-48 between CHN24001 and CHN24025. The red arrows represent mobile genetic elements (MGEs), the yellow arrows represent antimicrobial resistance genes, and the grey arrows represent open reading frames (ORFs).

Due to the closely related core genomic backgrounds of these isolates, we intended to explore the similarity of the plasmid profile in these isolates. The results of S1-PFGE and Southern blotting showed that *bla*_NDM-1_ and *bla*_OXA-48_ were located on the different plasmids among these isolates. The molecular sizes of plasmids that harboured truncated *bla*_NDM-1_ (pNDM-1) were essentially the same; however, the plasmids encoding *bla*_OXA-48_ (pOXA-48) seemed distinct, with pOXA-48 in CHN24001 and CHN24069 being smaller than that in CHN24003, CHN24025, and CHN24039 (Figure 2A).

To obtain an elaborate understanding of the plasmid structure, we performed long-read sequencing. The results showed that pNDM-1 was an IncA/C2 plasmid (149,808 bp), which had *ble*_MBL_, *rmtC*, *sul1*, *aac(6′)-Ib11*, *bla*_CMY-6_, and truncated *bla*_NDM-1_, and pOXA-48 was an IncL/M plasmid (62,546 bp), which only had *bla*_OXA-48_ (Figure 2B). The plasmid comparison showed that the IS*1SD* element-mediated insertion event made the pOXA-48 of CHN24003, CHN24025, and CHN24039 larger than that in CHN24001 and CHN24069 (Figure 2C).

### 2.4. Conjugation Assay and Gene Cloning Experiments

To understand the transmission ability of these plasmids, we performed a conjugation assay. The pOXA-48 transconjugants were obtained when CHN24039 was set as the donor, and the pNDM-1 transconjugants were obtained when CHN24001, CHN24025, or CHN24039 was set as the donor. For all the transconjugants harbouring pOXA-48 or pNDM-1, both the plasmids were believed to be transmissible between different bacteria. The AST results showed that either the pOXA-48 or pNDM-1 transconjugants were resistant to ertapenem, and even pNDM-1 had a truncated NDM-1, which was inactive, as previously presented (Table 2).

Then, we investigated the carbapenem resistance mechanism of transconjugants harbouring pNDM-1. We introduced *bla*_CMY-6_ to determine whether CMY-6 could elevate the MIC of ertapenem. The results showed that *bla*_CMY-6_ could elevate the MIC of ertapenem more than 64-fold in *Escherichia coli* DH5α, as well as 4–8-fold for imipenem and meropenem (Table 2).

## 3. Discussion

In this study, we identified five ST307 CRKP isolates harbouring *bla*_CMY-6_, *bla*_OXA-48_, and a truncated *bla*_NDM-1_. ST307, first reported in 2008, is now considered an emerging high-risk antimicrobial-resistant (AMR) clone [10] and is endemic in Italy, Colombia, the United States (Texas), and South Africa [7]. Previous research showed that ST307 strains contained *gyrA* and *parC* mutations, which aided in their global distribution [7].

ST307 is associated with various carbapenem resistance determinants, including KPC-2 and -3 [11], OXA-48 and -181 [12,13], NDM-1 [14], and VIM-1 [15]. The plasmid replicon types of pNDM-1 and pOXA-48 in this study were IncA/C2 and IncL/M, respectively. It has been revealed that IncL/M-type plasmids prefer to be associated with *bla*_OXA-48_ rather than any other additional ARGs [16], which was consistent with the results of this study. Moreover, *bla*_NDM-1_ has been observed on diverse plasmid types, such as the narrow-host incompatibility group IncF and the wide-host incompatibility groups IncA/C, IncL/M, IncH, and IncN [17].

Previous research indicated that the *bla*_CTX-M-15_ gene was present among 99% of ST307 isolates [18] and primarily located on the chromosome with two to three copies. However, *bla*_CTX-M-15_ was located on the IncF(II)K plasmid in this study and was absent in three of five isolates. The sequence comparison indicated that an additional ~46 kb MDR segment existed in CHN24001 and CHN24069, in which *bla*_CTX-M-15_, *aac(3)-IIe*, *catB*, *bla*_OXA-1_, *aac(6′)-Ib-cr6*, *tet(A)*, *qnrB1*, and *dfrA14* were dispersed between multiple IS*26*, IS*Ecp1*, and other insertion sequences (Figure S3). Based on the results for the truncated *bla*_NDM-1_ and the differences in pOXA-48 and pCTX-M-15 among these isolates, the insertion elements were associated with genomic instability in ST307, especially IS*1SD*, IS*10*, and IS*26*.

In this study, *bla*_NDM-1_ was disrupted by IS*10*, which belongs to the IS*4* family. According to Vila et al., the native signal peptide was associated with NDM-1 anchored to the outer membrane, which influenced the concentration of soluble NDM-1 in the periplasm [19], and IS inserted into the signal peptide of *bla*_NDM-1_ abolished the carbapenemase function of NDM-1, which was consistent with our results. The mechanism underlying carbapenem resistance in *K. pneumoniae* generally involves the production of carbapenemase; additionally, the production of AmpC and ESBL along with the loss or decreased expression of outer membrane proteins play an important role [20]. CMY is a class C β-lactamase, and *bla*_CMY-6_ is rare in *K. pneumoniae*. A previous study has shown that carbapenem resistance in *E. coli* can arise via high-level expression of CMY-4 [21], and another study showed that CMY-2 could make *E. coli* resistant to meropenem [22]. To the best of our knowledge, we are the first to report that *bla*_CMY-6_ can elevate the MIC of carbapenems.

## 4. Materials and Methods

### 4.1. Collection of Isolates and Bacterial Identification

The five CRKP isolates examined in this study were selected from a national observational, multicentre study in China, which included 28 hospitals around mainland China. This multicentre study was approved by the local ethics committees of Sir Run Run Shaw Hospital (20170301-3). The species of isolates were identified by matrix-assisted laser desorption ionisation–time of flight (MALDI-TOF) mass spectrometry systems (Skyray Instrument, Kunshan, China).

### 4.2. Antimicrobial Resistance Testing

The ASTs of imipenem, meropenem, ertapenem, ceftazidime, and aztreonam for all the isolates were performed following the standard protocol for the microbroth dilution method. *E. coli* ATCC 25,922 was used as the quality control, and the breakpoint was interpreted according to CLSI guidelines [23].

### 4.3. PFGE, S1-PFGE, and Southern Blotting

PFGE and S1-PFGE were performed using the contour-clamped homogeneous electric field (CHEF) mapper (Rio-Rad, Hercules, CA, USA) as previously described [24]. Briefly, the DNA was digested by *XbaI* for PFGE and digested by S1 nuclease for S1-PFGE. *Salmonella enterica* H9812 DNA digested by *XbaI* was used as a marker. Digoxigenin-labelled *bla*_NDM-1_ and *bla*_OXA-48_ probes (Roche Diagnostics, Basel, Switzerland) were used for Southern blotting, and the probe primers are listed in Table S1.

### 4.4. Whole-Genome Sequencing and Subsequent Analysis

The genomic DNA of all the isolates was extracted by a QIAamp DNA Mini Kit (QIAGEN, Hilden, Germany) and subjected to whole-genome sequencing on the Illumina HiSeq xTen platform (Illumina, San Diego, CA, USA) with a 150 bp paired-end strategy, as previously described [25]. Isolates CHN24001 and CHN24025 were further subjected to long-read sequencing by a MinION sequencer device (Nanopore Technologies, Oxford, UK) with a 1D flow cell. The short reads were de novo assembled by Shovill (https://github.com/tseemann/shovill (accessed on 12 June 2022)), and the long reads were assembled by Raven (https://github.com/lbcb-sci/raven (accessed on 22 July 2022)) and further polished by Polypolish using paired-end short reads [26].

The multilocus sequence type (MLST) and ARGs were identified by mlst (https://github.com/tseemann/mlst (accessed on 12 August 2022)) and ABRicate (https://github.com/tseemann/abricate (accessed on 12 August 2022)), based on the NCBI AMRFinderPlus database [27], respectively. The plasmid comparison was performed by Easyfig [28] and Proksee (https://proksee.ca (accessed on 15 August 2022)) and annotated by BacAnt and Prokka [29,30]. cgMLST was performed by Ridom seqsphere (version 6.0.0, Ridom GmbH, Münster, Germany), as previously described [31]. The randomly selected 92 isolates from different continents along with the 5 isolates in this study were imported for phylogenetic relationship analysis based on their single-nucleotide polymorphisms (SNPs) using Snippy [25]. The genome of C234 was used as a reference (accession number: SAMN15868954) [9], and the tree was illustrated by iTol (https://itol.embl.de (accessed on 22 August 2022)). The isolates selected from the public database are listed in Table S2.

### 4.5. Conjugation Experiments

Briefly, the rifampin-resistant *E. coli* strain EC600 served as the recipient cell, and the *K. pneumoniae* isolates served as donors. Rifampin (300 mg/L) and ertapenem (0.5 mg/L) were simultaneously used for transconjugant selection. The conjugative isolates were confirmed by MALDI-TOF and S1-PFGE. The ARGs of *bla*_NDM-1_ and *bla*_OXA-48_ were confirmed by PCR (Table S1).

### 4.6. Plasmid Construction

Gibson assembly was used for plasmid construction (Vazyme, Nanjing, China), and the primers are listed in Table S1. Since we aimed to study the β-lactamase, we first knocked out the ampicillin resistance gene on the plasmid pCR2.1 to form plasmid pCR2.1K. Then, *bla*_NDM-1_, truncated *bla*_NDM-1_, *bla*_OXA-48_, and *bla*_CMY-6_ were introduced into the plasmid pCR2.1K between the *NotI*/*ApaI* or *KpnI*/*BamHI* restriction enzyme sites.

### 4.7. Nucleotide Sequence Accession Numbers

The whole-genome sequencing (WGS) raw reads of *K. pneumoniae* isolates, derived by both Illumina and nanopore sequencing, were deposited under the project in the NCBI database (accession number: PRJNA875629).

## 5. Conclusions

In summary, the high-risk clone type ST307 CRKP was found in China, and these isolates all harboured *bla*_CMY-6_, *bla*_OXA-48_, and truncated *bla*_NDM-1_, which should be given additional attention. *bla*_CMY-6_ and *bla*_NDM-1_ were located on the IncA/C2-type plasmid, and *bla*_OXA-48_ was located on the IncL/M-type plasmid. NDM-1 was truncated by IS*10* at the signal peptide, which induced carbapenemase inactivity, and gene cloning of *bla*_CMY-6_ showed that it could induce carbapenem resistance.

## Figures and Tables

**Table 1:** The department, specimen type, ST, antimicrobial susceptibility, and beta-lactam resistance genes of *K. pneumoniae*.

Isolates	Department	Specimen Type	ST	MIC (mg/L) ^1^					Beta-Lactamases
				ATM	IPM	MEM	ETP	CAZ	
CHN24001	Department of Burns	Blood	307	>64	64	64	>64	>64	*bla*_NDM-1_^2^, *bla*_OXA-48_, *bla*_CMY-6_, *bla*_CTX-M-15_, *bla*_SHV-106_, *bla*_TEM-1_, *bla*_OXA-1_
CHN24003	Department of Burns	Secretion	307	>64	16	16	>64	>64	*bla*_NDM-1_^2^, *bla*_OXA-48_, *bla*_CMY-6_, *bla*_SHV-106_, *bla*_TEM-1_
CHN24025	Department of Burns	Secretion	307	>64	32	32	>64	>64	*bla*_NDM-1_^2^, *bla*_OXA-48_, *bla*_CMY-6_, *bla*_SHV-106_, *bla*_TEM-1_
CHN24039	Department of Burns	Secretion	307	>64	16	16	>64	>64	*bla*_NDM-1_^2^, *bla*_OXA-48_, *bla*_CMY-6_, *bla*_SHV-106_, *bla*_TEM-1_
CHN24069	Department of Hematology	Blood	307	>64	64	64	>64	>64	*bla*_NDM-1_^2^, *bla*_OXA-48_, *bla*_CMY-6_, *bla*_CTX-M-15_, *bla*_SHV-106_, *bla*_TEM-1_, *bla*_OXA-1_

^1^ ATM: aztreonam, IPM: imipenem, MEM: meropenem, ETP: ertapenem, CAZ: ceftazidime; ^2^ The *bla*_NDM-1_ gene was truncated.

**Table 2:** Antimicrobial susceptibility of conjugates and the gene cloning isolates.

Isolates	MIC (mg/L) ^1^				
	ATM	IPM	MEM	ETP	CAZ
Conjugation					
EC600	0.125	0.25	<0.03	<0.03	0.5
E24039J-pOXA-48	0.25	2	1	16	0.25
E24001J-pNDM-1	>64	1	0.25	2	>64
E24025J-pNDM-1	>64	1	0.25	4	>64
E24039J-pNDM-1	>64	1	0.25	8	>64
Gene cloning					
DH5α	<0.03	0.25	<0.03	<0.03	0.125
DH5α-pCR2.1K	0.0625	0.25	<0.03	<0.03	0.25
DH5α-pCR2.1K::NDM-1	0.0625	32	64	64	>64
DH5α-pCR2.1K::NDM-1(T) ^2^	0.125	0.25	<0.03	<0.03	0.5
DH5α-pCR2.1K::CMY-6	>64	1	0.25	2	>64
DH5α-pCR2.1K::OXA-48	0.25	2	0.5	16	0.5
DH5α-pCR2.1K::NDM-1(T) ^2^::CMY-6	>64	0.5	0.25	2	>64
DH5α-pCR2.1K::NDM-1(T) ^2^::OXA-48	0.25	2	1	8	0.5

^1^ ATM: aztreonam, IPM: imipenem, MEM: meropenem, ETP: ertapenem, CAZ: ceftazidime; ^2^ NDM-1(T) contained the IS*10* sequence at position 25 and residues of NDM-1.

## Data Availability

The raw reads of *K. pneumoniae* isolates were deposited under the project in the NCBI database (accession number: PRJNA875629).

## Supplementary Materials

The following supporting information can be downloaded at: https://www.mdpi.com/article/10.3390/antibiotics11111616/s1, Figure S1: (A) The PFGE pattern of CHN24001, CHN24003, CHN24025, CHN24039, and CHN24069. (B) Minimum spanning tree of CHN24001, CHN24003, CHN24025, CHN24039, and CHN24069 analysed by cgMLST. Figure S2: Schematic of truncated *bla*_NDM-1_ interrupted by IS*10*. The direct repeat (DR) franking the insertion was TGCTGAGCG, and the inverted repeats (IRs) of IS*10* close to the DR were CTGATGAATCCCCT and AGGGGATCTCTCAG. Figure S3: CTX-M-15-harbouring plasmid comparison among these isolates. The red ring represents CHN24001, the yellow ring represents CHN24003, the blue ring represents CHN24025, the brown ring represents CHN24039, and the green ring represents CHN24069. Antimicrobial resistance genes (ARGs) and mobile genetic elements (MGEs) labelled on the outer ring. Table S1: The primers used in this study. Table S2: The ST307 isolates worldwide used in the phylogenetic analysis.
