# Supplementary material for: The Genetic Characteristics and Carbapenem Resistance Mechanism of ST307 Klebsiella pneumoniae Coharbouring blaCMY-6, blaOXA-48, and a Truncated blaNDM-1
Source: Antibiotics (Basel). 2022 Nov 13;11(11):1616. doi: 10.3390/antibiotics11111616 (PMC9687000; doi:10.3390/antibiotics11111616)
Supplement: Supplementary file 1 [file antibiotics-11-01616-s001.zip › antibiotics-2004398-supplementary.pdf]

Figure S1 (A) The PFGE pattern of CHN24001, CHN24003, CHN24025, CHN24039, and CHN24069. (B) Minimum spanning tree of CHN24001, CHN24003, CHN24025, CHN24039, and CHN24069 analyzed by cgMLST.

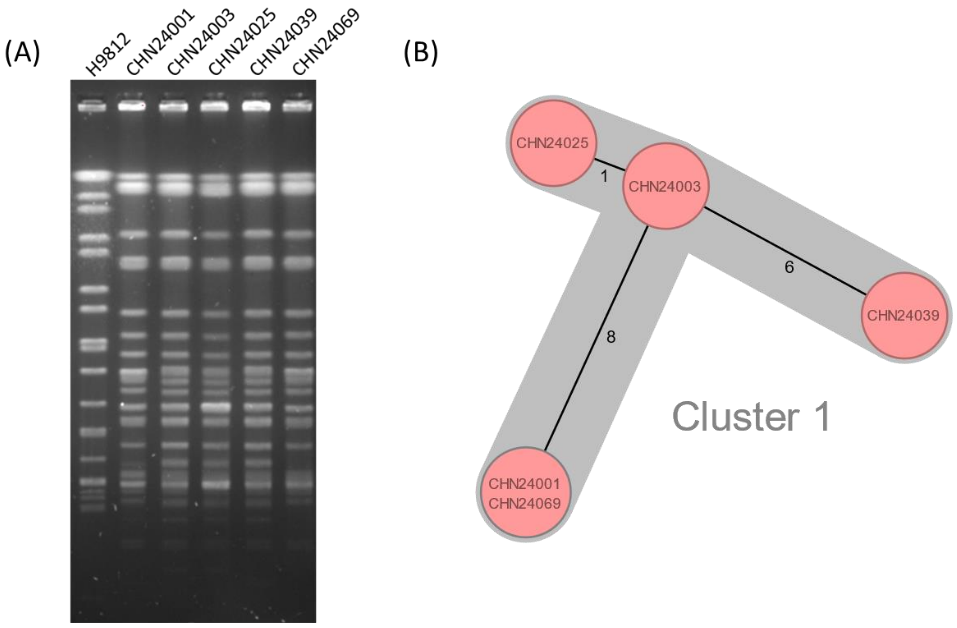

Figure S2 The schematic of truncated *bla*NDM-1 interrupted by IS*10*. The direct repeats (DRs) franked the insertion were TGCTGAGCG, and the inverted repeats (IRs) of IS*10* close to the DRs were CTGATGAATCCCCT and AGGGGATCTCTCAG, respectively.

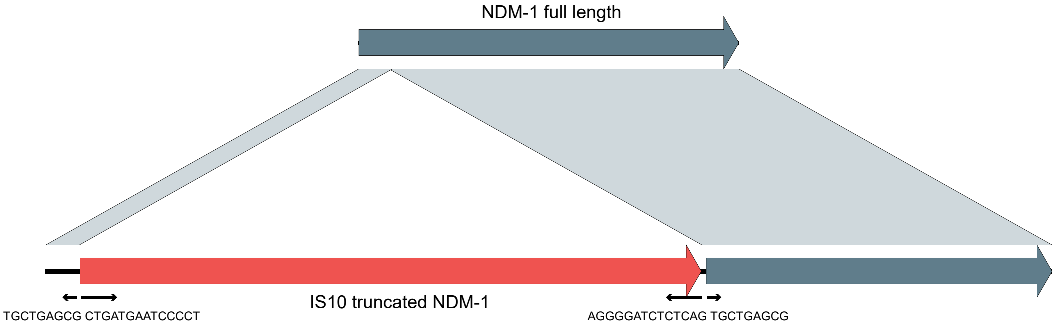

Figure S3 The CTX-M-15 harbouring plasmid comparison among these isolates. The red ring represents CHN24001, the yellow ring represents CHN24003, the blue ring represents CHN24025, the brown ring represents CHN24039, and the green ring represents CHN24069. Antimicrobial resistance genes (ARGs) and mobile genetic elements (MGEs) were labelled on the outer ring.

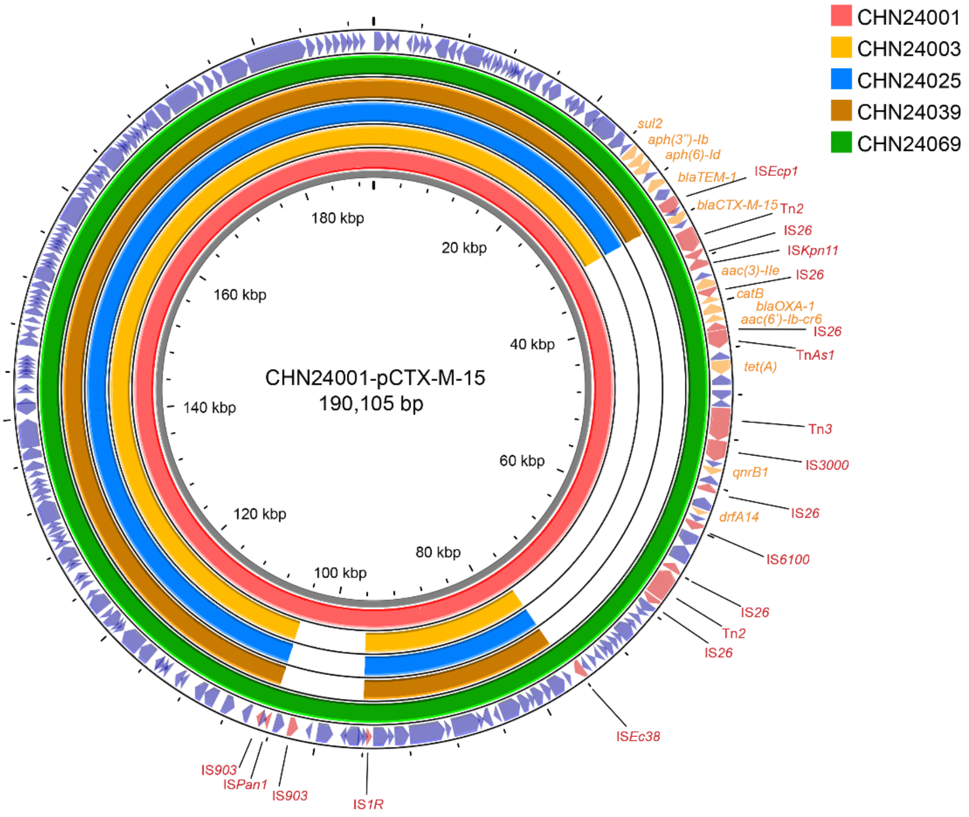

Table S1 The primers used in this study.

Primers	Sequence (5'-3')	Description
Southern Blot		
NDM-1-F-Southern	CGCAACACAGCCTGACTTTC	NDM-1 probe for southern blot
NDM-1-R-Southern	GGCGGAATGGCTCATCACGA	
OXA-48-F-Southern	GCGTGGTTAAGGATGAACAC	OXA-48 probe for southern blot
OXA-48-R-Southern	CATCAAGTTCAACCCAACCG	
Conjugation		
NDM-1-F-Validate	ATGGAATTGCCCAATATTATGCACCCGGTC	NDM-1 full length validation
NDM-1-R-Validate	TCAGCGCAGCTTGTCGGCCA	
OXA-48-F-Validate	ATGCGTGTATTAGCCTTATC	OXA-48 full length validation
OXA-48-R-Validate	CTAGGGAATAATTTTTTCCTGTTTG	
Gene cloning		
pCR2.1-del-AmpR-1	caggatgaggatcgtttcgcATGATTGAACAAGATGGATTGCAC	Amplification for kanamycin resistance gene
pCR2.1-del-AmpR-2	gagtaaacttggtctgacagTCAGAAGAACTCGTCAAGAAGGC	
pCR2.1-del-AmpR-3	CTGTCAGACCAAGTTTACTCATATATACTTT	Amplification for backbone of pCR2.1
pCR2.1-del-AmpR-4	GCGAAACGATCCTCATCCTG	
NDM-1-truncated-clone-F	atatccatcacactggcggccgcTAAATTGGAGTCATTACCAGATTGGC	Amplification for truncated NDM-1 gene and its
NDM-1-truncated-clone-R	actatagggcgaattgggcccTCAGCGCAGCTTGTCGGC	promoter
NDM-1-FL-clone-1	ggcaattccatCAAGTTTTCCTTTTATTCAGCATTAAA	Amplification for full length NDM-1 gene and the
NDM-1-FL-clone-2	gaaaacttgATGGAATTGCCCAATATTATGCA	same promoter to truncated NDM-1
CMY-6-FL-clone-F	gattacgccaagcttggtaccATGTTACAATGTGTGAGAAGCAGTCTAA	Amplification for CMY-6 gene and its promoter
CMY-6-FL-clone-R	gcggccgttactagtggatccTTATTGCAGCTTTTCAAGAATGCG	
OXA-48-FL-clone-F	gattacgccaagcttggtaccCCATCATACACTAAATCAGTAAGTTGGC	Amplification for OXA-48 gene and its promoter
OXA-48-FL-clone-R	gcggccgttactagtggatccCTAGGGAATAATTTTTTCCTGTTTGA	

Table S2 The ST307 isolates worldwide used in the phylogenetic analysis.

Isolates	Accession number	Region
EC03605938	GCF_015264255.1_ASM1526425v1	Africa
EC03607707	GCF_015264295.1_ASM1526429v1	Africa
EC03607709	GCF_015264315.1_ASM1526431v1	Africa
EC03612985	GCF_015264215.1_ASM1526421v1	Africa
EC03629993	GCF_015264225.1_ASM1526422v1	Africa
EC03632007	GCF_015594555.1_ASM1559455v1	Africa
G3435	GCF_002187305.1_ASM218730v1	Africa
I72	GCF_003934185.1_ASM393418v1	Africa
Kpn01733	GCF_902703005.1_Kpn01733	Africa
Q1445	GCF_903111795.1_CHZ10	Africa
C1287	GCF_003954965.1_ASM395496v1	Asia
CPKp171210	GCF_009760795.1_ASM976079v1	Asia
ESBL-SIUK-7	GCF_002239875.1_ASM223987v1	Asia
P169	GCF_003954015.1_ASM395401v1	Asia
P781	GCF_003954605.1_ASM395460v1	Asia
R2476	GCF_003955155.1_ASM395515v1	Asia
R3643	GCF_003953995.1_ASM395399v1	Asia
ST307PT02	GCF_003597715.1_ASM359771v1	Asia
ST307PT03	GCF_003597735.1_ASM359773v1	Asia
WCHKP080018	GCF_003261645.1_ASM326164v1	Asia
EuSCAPE_BE031	GCF_900506285.1_19506_1_10	Europe
EuSCAPE_ES138	GCF_900501105.1_18623_1_124	Europe
EuSCAPE_ES149	GCF_900501135.1_18623_1_135	Europe
EuSCAPE_FR035	GCF_900510505.1_17870_2_73	Europe
EuSCAPE_RO055	GCF_900504075.1_19084_3_140	Europe
ISMETT ST307 cl5	GCF_015321515.1_ASM1532151v1	Europe
Kpn17	GCF_015643815.1_ASM1564381v1	Europe
kpneu006	GCF_900607435.1_kpneu006	Europe
Kpngiani7132586	GCF_902704005.1_Kpngiani7132586	Europe
Kpngiani7132589	GCF_902703845.1_Kpngiani7132589	Europe
CRK0019	GCF_002186005.2_ASM218600v2	North America
CRK0072	GCF_002186255.2_ASM218625v2	North America
CRK0080	GCF_002185575.2_ASM218557v2	North America
CRK0135	GCF_001902865.4_ASM190286v4	North America
CRK0353	GCF_002936395.2_ASM293639v2	North America
CRK0363	GCF_002935385.2_ASM293538v2	North America
CRK0372	GCF_002935665.2_ASM293566v2	North America
CRK0379	GCF_002935585.2_ASM293558v2	North America
KCJ3K379	GCF_008245305.1_ASM824530v1	North America
KLP00218	GCF_012952365.1_ASM1295236v1	North America
5R	GCF_003194695.1_ASM319469v1	South America
CCBH26672	GCF_016056175.1_ASM1605617v1	South America

CCBH27755	GCF_016055435.1_ASM1605543v1	South America
CCBH27869	GCF_015666615.1_ASM1566661v1	South America
GMR-RA-453.16	GCF_002283495.1_ASM228349v1	South America
preta	GCF_003194705.1_ASM319470v1	South America
ARLG-3198	C252	USA: Houston
ARLG-4260	C4691	USA: Houston
KPN1022	SRR5385804	USA: Houston
KPN1109	SRR5385730	USA: Houston
KPN1329	SRR5386846	USA: Houston
KPN1333	SRR5386842	USA: Houston
KPN1388	SRR5386789	USA: Houston
KPN1390	SRR5386787	USA: Houston
KPN1499	SRR5386673	USA: Houston
KPN1595	SRR5386132	USA: Houston
KPN1598	SRR5386129	USA: Houston
KPN161	SRR5386427	USA: Houston
KPN1634	SRR5386106	USA: Houston
KPN1651	SRR5386095	USA: Houston
KPN1677	SRR5386069	USA: Houston
KPN1678	SRR5386068	USA: Houston
KPN1687	SRR5386061	USA: Houston
KPN1723	SRR5386025	USA: Houston
KPN1761	SRR5385976	USA: Houston
KPN182	SRR5386412	USA: Houston
KPN1868	SRR5387398	USA: Houston
KPN1894	SRR5387374	USA: Houston
KPN1925	SRR5387353	USA: Houston
KPN1999	SRR5387294	USA: Houston
KPN2027	SRR5387268	USA: Houston
KPN2038	SRR5387258	USA: Houston
KPN2083	SRR5387217	USA: Houston
KPN2086	SRR5387214	USA: Houston
KPN2098	SRR5387203	USA: Houston
KPN280	SRR5386324	USA: Houston
KPN287	SRR5386317	USA: Houston
KPN336	SRR5386271	USA: Houston
KPN347	SRR5386262	USA: Houston
KPN354	SRR5386258	USA: Houston
KPN364	SRR5386248	USA: Houston
KPN485	SRR5385515	USA: Houston
KPN515	SRR5385503	USA: Houston
KPN575	SRR5385452	USA: Houston
KPN578	SRR5385449	USA: Houston
KPN611	SRR5385420	USA: Houston

KPN859	SRR5386473	USA: Houston
KPN863	SRR5386469	USA: Houston
KPN877	SRR5385931	USA: Houston
KPN913	SRR5385899	USA: Houston
KPN925	SRR5385888	USA: Houston
KPN940	SRR5385873	USA: Houston
CHN24001	SAMN27864414	This study
CHN24003	SAMN27864415	This study
CHN24025	SAMN27864421	This study
CHN24039	SAMN27864422	This study
CHN24069	SAMN27864428	This study
